# Understanding the Support Needs of People With Long COVID: A Qualitative Study in Aotearoa New Zealand

**DOI:** 10.1111/hex.70965

**Published:** 2026-11-09

**Authors:** Fiona McKenzie, Bailey Yee, Rochelle Ellison‐Lupena, Ridvan Firestone, Lis Ellison Loschmann, Kim van Wissen, Mona Jeffreys

**Affiliations:** ^1^ Te Puna Hauora, School of Health Te Herenga Waka‐Victoria University of Wellington Wellington New Zealand; ^2^ Flax Analytics Ltd Wellington New Zealand; ^3^ Centre for Public Health Research Massey University Wellington New Zealand

**Keywords:** access to healthcare, co‐design, lived experience, Long COVID, participatory research, post‐COVID condition, primary health care

## Abstract

**Introduction:**

Long COVID is a multi‐system condition that affects an increasing number of individuals. Little is known about the support needs of people living with long COVID in Aotearoa New Zealand. This study aimed to explore the lived experiences and healthcare access challenges of people with long COVID to better inform service design.

**Methods:**

Co‐design methodology was used, informed by the Stanford Design Thinking process and knowledge mobilisation approaches. Participants were recruited via snowballing, through professional networks and support groups. People with lived experience of long COVID were invited to participate in a 2‐h workshop, facilitated by researchers, conducted between March and September 2024. Participants were asked to create a depiction of their experience of long COVID and describe their creation to the group; descriptions were audio‐recorded, transcribed and thematically analysed. The inclusion of people with lived experience of long COVID as co‐investigators ensured that the study was designed and conducted to forefront their knowledge and experience.

**Results:**

Eight workshops were conducted, including 30 participants, between March and August 2024. Of these, 25 (83%) were female; of the 25 who provided their age, most were aged 45 to 64 years (*n* = 18, 72%), four (16%) were aged 25 to 44 years and three (12%) aged 65 years or older. Five key themes emerged: (i) lack of assistance; (ii) systemic barriers; (iii) absence of long COVID clinics; (iv) financial strain; and (v) low quality of care. A sixth theme identified personal coping strategies.

**Conclusions:**

Individuals with long COVID in Aotearoa New Zealand face major barriers to care, including inadequate services, financial hardship, and fragmented healthcare pathways. Findings support improved pathways and primary health care education in long COVID. A person‐centred, coordinated, and accessible model of care is needed.

**Lived Experience or Public Contribution:**

The study was designed with two people with lived experience of long COVID included as co‐investigators, and a further one on the Advisory Board. All participants had lived experience of long COVID.

## 1 Introduction

Long COVID, also known as Post‐COVID condition, is defined variously by different entities (Greenhalgh, 2024), but in Aotearoa New Zealand, the clinical definition is COVID‐19‐related ongoing symptoms that continue 12 weeks or more after infection with SARS‐CoV‐2 [1]. The term ‘long COVID’ encompasses a myriad of symptoms with a full spectrum of severity from mild and transient symptoms to life‐limiting conditions [2, 3, 4]. Common ongoing symptoms such as fatigue or general malaise, cognitive impairment and respiratory symptoms have a substantial impact on quality of life, often requiring significant lifestyle changes [3, 5]. However, the pathophysiology underlying long COVID remains unclear [6] and there is currently no evidence‐based treatments or cure [5].

The global pooled prevalence of ever having long COVID has been estimated at 36% (95% confidence interval, 33% to 40%) from 144 contributing studies [7]. Recently, the New Zealand Health Survey estimated that 9% of adults reported ever having had long COVID symptoms [8], with half (49%) still experiencing symptoms at the time of being interviewed. The lower prevalence in Aotearoa New Zealand compared to other countries is likely due to the very low incidence of COVID‐19 in pre‐Omicron phases of the pandemic, as well as very high vaccination rates prior to COVID‐19 becoming endemic in Aotearoa New Zealand.

In many developed countries, long COVID clinics were the mainstay of care, such as in the United Kingdom (UK), where the National Health Service (NHS) provided significant investment to expand long COVID services beyond Post‐COVID Assessment Clinics to strengthen diagnostics, treatment and rehabilitation [9]. In Aotearoa New Zealand, however, the burden of long COVID management has largely fallen to primary health care. This has created barriers to care for many people; in Aotearoa New Zealand, most people pay a co‐payment to access primary health care. Furthermore, access to specialist care requires a referral from a general practitioner (GP) and can often be associated with long waiting lists or the refusal of a specialist to see a patient. Seeing a specialist privately is possible for people with health insurance, or those able to pay considerable out‐of‐pocket costs.

Early in the pandemic, and in the absence of publicly available services, a group of patients identified the need for a patient support group and established Long COVID Support Aotearoa [10]. This has evolved into one of the key peer support groups for people with long COVID. Website content is written by people with long COVID, and fact‐checked by health professionals with long COVID. Patient and public involvement (PPI) was implemented in this study through close involvement with this support group; we included people with lived experience of long COVID as co‐investigators and advisors in our research, we centred the experiences and knowledge of patients in this work.

The aim of this study was to document the experiences of people with long COVID in Aotearoa New Zealand in accessing support, with a focus on primary health care services.

## 2 Materials and Methods

The study was the first phase of a larger long COVID project, entitled ‘Evidence‐based Management of Long COVID’ [11]. People with lived experience of long COVID were included as co‐investigators and advisors and thus were central to the design and conduct of the research. The part of the work described here was underpinned by co‐design methodology [12], with the explicit aim to place patients’ experiences at the centre of the design process. We used a five‐phase approach informed by the Stanford Design Thinking process [13]. The phases are (i) empathise; (ii) define; (iii) ideate; (iv) prototype; (v) test. We report here on the ‘empathise’ and ‘define’ phases, which focus on understanding participants’ lived experiences and identifying common barriers to care that people with long COVID had identified. For these initial phases we drew on participatory co‐design methods [14, 15], given a key focus of the research was including participants as co‐creators in developing possible support pathways or primary health care service interventions for people with long COVID. In the later phases (to be described in a subsequent publication), the barriers were consolidated during the ‘ideate’ phase into a set of prototypes. During the ‘prototype’ phase, the ideas were considered and discussed with participants in iterative workshops, where they were refined into a final prototype. The ‘testing’ phase of the process was outside the scope of the funded project.

Participants with long COVID were identified through various sources, including snowballing via our ongoing programme of long COVID research, the Long COVID Support Aotearoa group, and other professional networks. Potential participants were asked to contact the research team to express an interest in the study, following which the Participant Information Sheets were sent directly by the research team by e‐mail, with an invitation to co‐design workshops. These were scheduled across urban centres, as well as having an online option for those in other areas, and/or those too unwell to travel to face‐to‐face workshops. Māori, the Indigenous people of Aotearoa New Zealand, were invited by Māori researchers to Māori‐specific workshops, and Pacific peoples were invited to Pacific‐led workshops, the results of which will be disseminated separately. We report here on the non‐Māori/non‐Pacific workshops and data only.

Each workshop lasted approximately 2 h with a break part way through; the virtual workshops were conducted in two 45‐min sessions to minimise fatigue. At each workshop, a range of materials including paper, pens, cardboard and coloured tape were available for participants to use, if they chose, to facilitate the sharing of their stories through words, story or collage as a visual representation of their experience of long COVID and the support that they received, or did not receive but would have liked to receive. Materials were posted in advance to participants in virtual workshops.

All participants elected to make use of the provided materials while engaging in conversation about their long COVID journey with the facilitators (members of the research team) and each other. The workshops were guided by a prompt question, that was available electronically or in paper throughout the activity: ‘Create something with the materials that describes your experience of living with long COVID, and your support systems’. Approximately halfway through the workshop, participants were asked to share their written or visual creations with the rest of the group. The workshops were audio‐recorded and transcribed.

Transcripts were entered into Nvivo and coded thematically by one researcher (BY) using a general inductive approach [16]. A second researcher (REL) was given segments of the raw text from which some of the initial codes were developed and, without seeing the categories, was asked to create a second set of codes from the raw text [17]. Discrepancies were discussed with the analysis team. To further improve the validity of our analysis process, a third researcher (KvW) was provided with the codebook, which contained descriptions of the codes and the raw data associated with them. She thoroughly reviewed the codebook and coded text to ensure that the beginning stages of theme generation met a high‐quality standard. Themes and subthemes were then finalised by the full team. Preliminary themes were presented in a virtual feedback workshop to participants who had previously attended one of the workshops, seeking feedback to assess and confirm research findings and interpretations.

Informed consent was obtained from each participant prior to the workshop. Ethical approval for the study was given by Victoria University of Wellington Human Ethics Committee 2023/HE312492. The GRIPP2 long form checklist [18] was used to guide the reporting of our results.

## 3 Results

We conducted eight workshops, including 30 non‐Māori/non‐Pacific people between March and August 2024. Six workshops were conducted face‐to‐face and two were conducted virtually. A further workshop only included support people or carers; analysis of their data did not identify any themes that had not been identified by people with long COVID, so this data is not included here.

Of the 30 participants, 25 (83%) were female; of the 25 people who provided their age, most were aged 45 to 64 years (*n* = 18, 72%), four people (16%) were aged 25 to 44 years, and three (12%) aged 65 years or older. Examples of the artwork produced, with some accompanying quotes, are shown in Figure 1.

**Figure 1:**
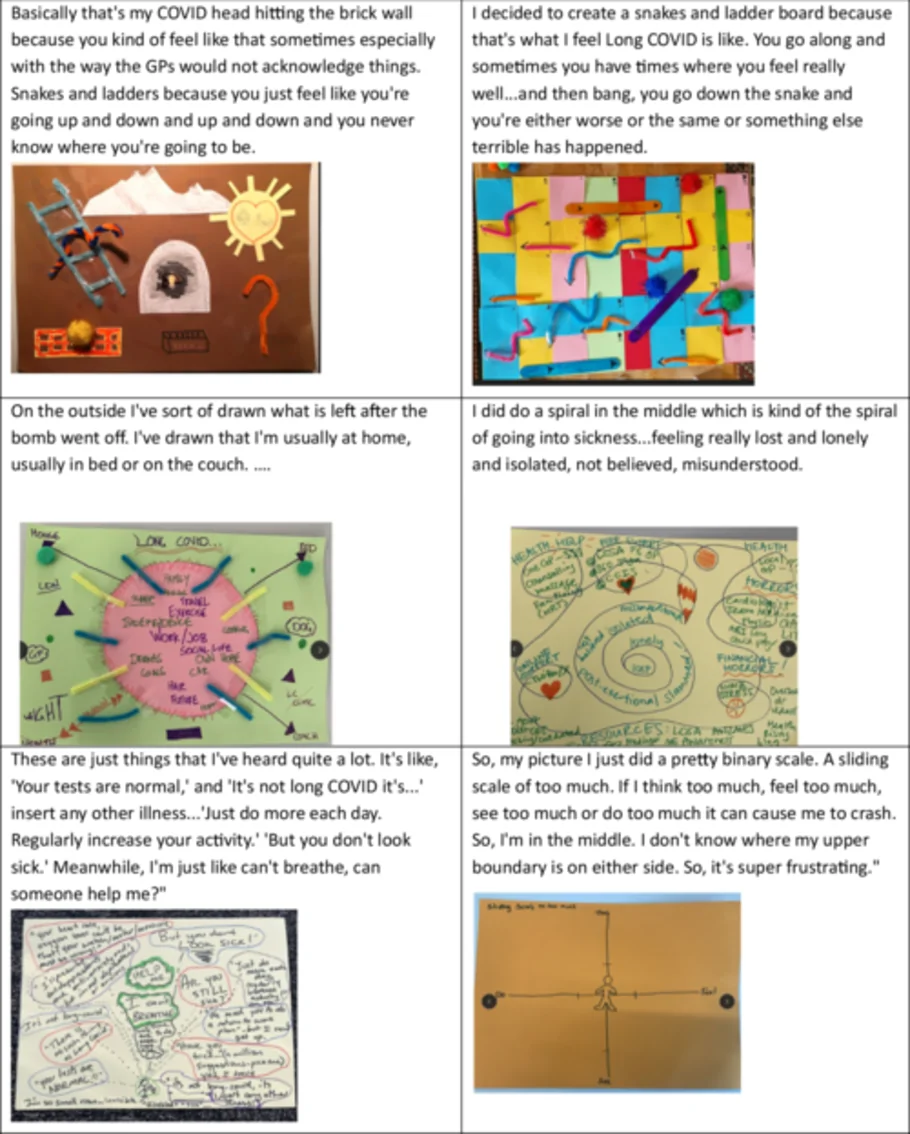
Examples of craft creations shared from workshop.

Approximately 200 preliminary codes were developed upon initial analysis. Preliminary codes were further condensed down to approximately 95 unique codes after completing all analyses of workshop transcripts, and then further collapsed to 27 codes. Five key interconnected and overlapping themes emerged from the analysis: lack of support; systemic barriers to accessing care; the absence of long COVID clinics; financial strain; and low‐quality care. A sixth theme described the strategies participants used or devised to cope with their long COVID journey, in the absence of appropriate support from the health system. No changes to these themes were suggested by participants during the virtual feedback workshop.

### 3.1 Lack of Support

The lack of support the participants experienced encompassed all facets of their lives. Feeling understood, heard, validated and reassured by health professionals was vitally important to participants. A recurring experience was that of not being believed, by the medical profession, by the public, and by family and friends. Most participants had average‐to‐high health literacy, and some had a medical/health background that supported them being able to identify needs and seek healthcare.

I felt this overwhelming sense in the beginning of reaching out, wanting people to help me, but they weren't there, and it was really horrible. You know, like the doctor wasn't there, the people who I thought would be sympathetic didn't get it, so there's this sense of just reaching out in frustration.

[P2N]

Within the health system, participants described an absence of coordinated care, medical guidance, or emotional support. This need for holistic care was identified, given the range of symptoms of long COVID, and an inability of the health system to provide this. As a consequence, participants spoke about being vulnerable, scared and exhausted from trialling alternative remedies that fail to improve health outcomes, compounded further by cognitive difficulties and ‘brain fog’ which affected most participants.

I did go to my doctor and she was really lovely but completely unhelpful because she didn't know what to advise, because I think what happens in the medical profession is they get very siloed within their own particular speciality, and so you're talking about something which is holistic, where you're getting stomach problems and bladder problems and headaches and exhaustion.

[P2N]

Participants felt frustration that the government‐recommended rehabilitation approaches to care were only available at the individual's expense. They wanted targeted funding and long COVID recognised as a disability to aid access to funded disability support services; they spoke about the feeling of being left behind. General awareness surrounding long COVID needs to be increased, so they did not face stigma or belittlement of their experiences.

My GP tracked me throughout the journey, and I think the problem is she wanted, ‘We're going to do some work with an occupational therapist,’ and we're still waiting to get that, apart from if you want to go private and it's like … $300 an appointment?

[P1N]

I think New Zealand's way behind because I know for a fact in the UK, where I'm from, they do a lot of alternative therapies for long Covid. They'll pay for somebody to go to acupuncture for this amount of weeks. If it's doing any good they keep with it. If it's not they change to something else. It makes me feel really jealous really and sad that we're all people, we all deserve the best, so why do we get nothing here?

[P2NP]

Many participants struggled with advocating for themselves, while coping with debilitating fatigue. Participants spoke of their continuous efforts to educate health professionals, and their lack of support across health and other services.

My medical centre has lots of [staff] turnover, so I almost had to educate each new person or find validation again. Hard when you don't look sick and have to grovel for support, understanding and advocacy.

[P2P]

### 3.2 Systemic Barriers to Accessing Care

The lack of support that participants felt from their health professionals was framed by some as a system‐wide problem. Participants expressed the lack of recognition from the government impacted the acceptability for them to seek healthcare and the acceptance from health professionals. This further impacted eligibility to seek financial aid, extended sick leave, and access to anti‐viral drugs.

Am I going to get better? I don't know because nobody has actually tried to figure out what's going on with me because my symptoms aren't severe enough to do anything except to disable me. But then, the government doesn't recognise it as a disability.

[P2CBD]

But, imagine if there was some funding there, facilitation from the government to help [Long COVID Support Aotearoa group] keep going. I have talking to people in primary care about where there's a shortage of nurses and GPs so you can't see patients one on one, could they tap into this group way of passing on education to groups of patients. Yes, you can't assess individuals for their individual needs but could they tap into that new way of working with people?

[P3V1]

One participant spoke about their GP expressing frustration with the constraints of the healthcare system, with specialists able to refuse to accept referrals. Others who were successful in getting a referral commented on the lack of care provided by specialists also.

I am lucky in that my doctor is quite open… I've just started on Low Dose Naltrexone and she was very open to prescribing that for me and going through the process where I know some people can't get their doctor to even acknowledge that's potential that people could try as a treatment. I know she's frustrated because we tried to get me in to see the team I think at [hospital name] and they just flatly refused because my heart ECG looked completely normal… I know that she's frustrated for that but I am lucky that I've got support [but] I think she does feel constrained by the system.

[P3V2]

It's been over a year ago now. I won't go back to the hospital to be honest because there's just no point. If I need to I just won't because there's no point.

[P2NP]

Participants often compared their experience of receiving support to other health conditions, such as cancer. They outlined the difference in the provision and timeliness of care as medical professionals had treatment pathways, medical testing that showed abnormalities and symptoms that were not dismissed.

I had a breast cancer diagnosis a few months after I'd been crippled with the long COVID, and so I was kind of going through two different medical systems, and I can tell you they are polar miles apart … these two treatment avenues were just so, so different.

[P1NP]

### 3.3 The Absence of Long Covid Clinics

For many participants being able to access a long COVID clinic that specialises in this condition would be the ideal. Some participants spoke about one clinic that was briefly open but closed due to inadequate funding. Although grateful for the service, they described their frustration with the lack of continuity of care at the clinic. Others described a disappointing experience at a different long COVID clinic, describing overwhelming amounts of written information being provided, which was inaccessible to patients with cognitive dysfunction.

I went into it thinking great, finally a long COVID Clinic. It might be a short‐term thing but hopefully they'll realise it's good and they'll keep it going. Literally from the first 5 min it was like, this isn't going to continue. This is literally just to use up some money.

[P2C]

For the long COVID clinic giving you bundles and bundles of stuff to read is not right because they should know that… giving somebody a pile of paperwork like that and then say, ‘Go and read it,’ they should know better.

[P2NP]

For one participant who was able to travel to an Australian long COVID clinic, the experience was positive, despite having to pay for the care she received.

It was extraordinary, because you go in there and that's their job, is to assess and work with you with long COVID. So, I'm privileged for being able to afford to do that.

[P4N]

Participants who had not been able to access specialised clinics identified a need for knowledgeable primary health care professionals, in particular with reference to post‐exertional malaise. This highlights ongoing need for education among health professionals working with long COVID patients.

They were quite empathetic but just no knowledge. It was like, I'm very sorry to hear that. That must be really hard for you. Just pace yourself and here are a few links to the Facebook group.

[P2P]

I was trying to explain what was happening with post exertional worsening which is totally my main and worst symptom, and she just didn't have a clue. It was like she just thought that I didn't want to do any exercise which is quite far from the truth.

[P3P]

### 3.4 Financial Strain

Affordability and financial implications surround all aspects of the long COVID journey, which was a key theme. Primary health care co‐payments for the multiple health visits and tests, and their associated physical and financial costs took great tolls on participants. Participants spoke of spending hundreds‐to‐thousands of dollars on supplements, vitamins, other alternative remedies, and private healthcare. Participants spoke of reducing or stopping some therapies entirely due to cost, or of stretching out medications to make them last for longer. Concern was expressed with the amount of money spent given their limited finances; some were in debt as most were willing to pay extensive amounts to receive some sort of relief from symptoms.

This [indicating poster] is my pile of vitamins and I have no idea what's working…Very expensive. And trial and error, and just going into it with a feeling of desperation.

[P3CBD]

I tried going off them [medications] just because of the cost and then I've gone into immediate crashes which have lasted for sort of 6 weeks. It's just not worth it. When I have a crash I can't speak, I have really bad balance.

Across all workshops participants outlined the difficulty in accessing financial assistance (i.e. government benefits from Work and Income NZ); many were unable to work due to their long COVID symptoms. They described the process as stressful, demeaning and physically and emotionally exhausting. There was a feeling that only those who had the energy to fight for the need for financial support received it.

Having to go to WINZ [Work and Income NZ] and say I can't work anymore and really feeling like, I was quite shocked at how I just felt like I was just a number. I wasn't important. [They implied that] surely I could be working if I really wanted to. Things like getting the accommodation supplement. I sold my home, my apartment. I moved in with my parents … because I've got a little bit of money left over from the sale of my apartment I'm not entitled to accommodation [supplement].

[P2C]

I just found the whole process of getting [government benefits] really stressful and just physically exhausting and emotionally exhausting… I don't know what the official policy situation is with that but to me it seems a bit uneven and it seems like the people who are willing and able to advocate for themselves or to get an MP involved, they're the ones who manage to get through to the permanent benefit.

[P3P]

### 3.5 Low‐Quality Care

There were recurring themes of inadequacy and low quality of care received, as well as a gap between services offered and participants’ needs. Some participants spoke about health professionals dismissing them or disregarding their symptoms, attributing them to psychological rather than physical causes. Other mentioned the lack of knowledge of GPs, and the need to identify possible treatments to suggest to their health professionals, rather than the other way around.

People said it's a psychological thing in your mind.

[P5C]

[Following referral to a neurologist] I got forwarded a copy of a letter and he said, ‘I think she needs clinical psychology and antidepressants.’ So I had to go back to my GP and he's sort of like hopping from one foot to the other feeling very awkward, because I don't think he didn't know what to say. He's not a neurologist. He goes, ‘I don't really think that will be helpful for you.’ But, he didn't really know what else to say. So he sort of said, ‘Sometime life is tricky’.

[P2P]

My GP didn't know anything. I found it really exhausting trying to do the research to then bring it to the GP to ask for something. I wished that didn't have to happen.

[P3V1]

Some participants reported that their health professionals had failed to further investigate symptoms, or to tailor services to fit their limitations. This was particularly in relation to people who suffered from post‐exertional malaise, who were often encouraged to pushed themselves physically, resulting in a worsening of symptoms.

Physiotherapy came a few times. Everything they got me to do … I would be in bed for about 4 days recovering. I was trying to explain about graded exercise and [that it] wasn't appropriate. Really nice person but message not getting through.

[P2V1]

### 3.6 Coping Strategies

Participants described a variety of strategies that they had adopted to cope with living with long COVID. These included reliance on family/whānau and friends; reaching out to support groups or services; and relying on their own research and advocacy. Of note, not all participants’ networks were supportive, reliable, or continuous, especially after extended periods of time. Some participants relied on family to physically access services, and others relied on family to pay for services due to their inability to work.

I'm living with my ex who takes care of me, which is an even more complicated situation. And yes, she has to do everything. She's really good about it, but she actually asked me to go out and get some help, some funding and some help, because she's working and studying and taking care of me, and it's just not fair on the people that we are lucky enough to have in our life. It just means that you rely on one person and if anything happened to them what would you do?

[P2CBD]

Last year I was in Australia doing agency work and then I just haven't worked since. I got Covid again once and fled home. I'm 28 and I'm living at home because I can't really manage by myself.

[P1V2]

However, not all participants were well supported by their social network. In some cases, there was a lack of support from friends, family, and people close to them. This, coupled with the lack of professional support, culminated in an overwhelming sense of isolation.

…losing health and also losing friends. The friends that I do have, I think they think I'm a hypochondriac.

[P5C]

Support groups provided both positive and negative experiences. The Long COVID Support Aotearoa Facebook page was heavily mentioned across all workshops, as it allowed participants to share experiences, find validation, and provided a platform to share alternative services, supplements or vitamins that helped or otherwise. While offering much needed validation, they often left the users feeling overwhelmed, anxious, and even despondent with no end in sight.

I joined Long COVID Aotearoa, the closed Facebook group and that was a revelation. Suddenly here's a community totally understanding what I'm going through.

[P3V2]

I joined up with Complex Chronic Illness Support, CCIS…That's been a fantastic thing for me and I accessed some online counselling through them.

[P1P]

## 4 Discussion

The aim of this qualitative study was to explore the needs of people living with long COVID and their experiences of accessing care in Aotearoa New Zealand. Study participants reiterated an overwhelming lack of support and lack of services for people with long COVID. Findings highlighted absence of pathways into primary or through to secondary care systems and it was emphasised that the current systems are not working for people with long COVID. The need for health professionals to gain additional education was emphasised. People living with long COVID expressed the need for coordination and continuity of care, including wrap‐around services due to the debilitating nature of the condition. Those with long COVID are currently forced to rely on friends and family for support, Facebook or charitable groups, and rely on themselves for advocacy and research into treatment options. There are huge financial implications for long COVID sufferers beyond the cost of care.

This study further highlights the difficulties of those living with long COVID in Aotearoa New Zealand [19], encompassing physical, cognitive, emotional, and financial hardship. By design, the study centred lived experience of long COVID. This enactment of PPI is as key strength of research. With two co‐investigators and one study advisor having experience of long COVID, patient involvement was key to the design and execution of the study, as well as the interpretation of the results. A further strength of the study design was the inclusion of both in person and virtual focus group workshops to accommodate those not able to leave their homes. Workshops were also held in various locations around the country to make them as accessible as possible. Nevertheless, despite efforts to support wide participation, people with severe long COVID were precluded from taking part due to their illness. Furthermore, participants predominantly suffered from symptoms of fatigue, cognitive dysfunction and pain. Other long COVID manifestations were less well represented, which may have biased the focus of our findings.

Arts‐informed approaches are increasingly being recognised in qualitative research as offering another way in which people can make sense of their lived‐experiences through different forms of expression such as literary techniques, performance and visual arts (Butler‐Kisber, 2010; [20]). In this study, the approach was a strength of our research, enabling all participants to engage meaningfully through the use of mixed words and representations of their experiences or feelings, and some used only artistic representations. Butler‐Kisber and Poldma [21] identified three attributes of collage in qualitative research: ‘as a reflective process, as a form of elicitation, and as a way of conceptualising ideas’ (p. 3). Those who took part in virtual workshops had often created more detailed artworks, as they did this in their own time. Both in person and virtual workshop participants told us that they found the experience useful, and many were proud of their creations, and opted to take them home. As researchers, we reflected that the methods used allowed participants to talk about their experiences in a way that placed the focus on the creative work rather than the individual, which facilitated talking about difficult situations and experiences.

Our findings closely mirror previous international research in this area [22, 23, 24] (Nelson, 2022) [25], as well as from Aotearoa New Zealand [19]. Feeling understood, heard, believed, validated and reassured was vitally important to our participants. This has been highlighted in international long COVID studies [23, 24, 26] and reinforces the need for greater understanding from both health professionals and the wider community [27]. A lack of belief or empathy from employers and health professionals has been found to contribute to the sense of burden and isolation among those with long COVID [24, 26, 27, 28, 29]. Participants often felt dismissed, disbelieved and gaslit, and that they had to prove their symptoms [19, 23, 24, 28, 29, 30]. It was to avoid these experiences influencing our research that we centred PPI in the design of our study.

In line with our findings, previous studies found participants wanted more clearly defined guidance on what they should be doing to help improve long COVID symptoms [23, 24, 31]. Research suggests pacing or activity management are helpful approaches while graded exercise is detrimental, leading to crashes and symptom exacerbation [23, 28, 30, 32].

Barriers to accessing healthcare found in this study reflect those described in international studies including unwillingness to refer, a lack of continuity of care and the lack of understanding or treatment options for long COVID [27, 28, 29]. Another common finding was that almost all participants felt they needed to self‐advocate to access services [19] (Brehon, 2023). Macpherson et al. [24] and Ladds et al. (2022) noted their study participants felt like they had to construct their own care pathways and advocate for themselves; and the need to self‐advocate was amplified if diagnostic assessments led to inconclusive results.

Despite the ending of COVID‐19 as a public health emergency in May 2023, SARS‐CoV‐2 remains endemic across the world, with new variants continuing to emerge. Given the long duration of illness for many people with long COVID, and the ongoing risk of long COVID following COVID‐19 illness, long COVID prevalence is unlikely to reduce. Recent data from Aotearoa New Zealand indicate a prevalence of 4.3% of self‐reported ongoing long COVID symptoms [33].

There is growing international evidence that focuses on rehabilitation and the mitigation of symptoms for those living with long COVID. Research that has focused on experiences accessing healthcare services for those with long COVID, their caregivers and GPs have come to similar findings. One common finding has been the need for improved GP empathy, improved knowledge of long COVID symptoms and treatment options available [34, 35, 36, 37]. UK based studies looking at GPs’ perspectives found that there was a lack of information about treatment and rehabilitation pathways or means of tracking treatment progression for patients, leading to hesitation to diagnose [38, 39]. It was also found that those with long COVID often exerted high levels of energy due to the need to self‐research and self‐advocate for treatment and rehabilitation options [23, 24, 40]. This has meant there has been inequitable access of rehabilitation care, as only those with the energy and the ability to advocate for, and afford these services have accessed them [39].

The affordability of care and support for people with long COVID was a dominant theme. In addition to the cost of healthcare, there are alternative treatments, therapies, and supplements that those living with long COVID trial in the absence of few evidence‐based treatment options. Many participants were unable to work due to their long COVID symptoms, as has previously been demonstrated [23, 41] amplifying financial pressures and stress. Furthermore, most were unable to access financial assistance such as income support or disability payments. Only one participant had the means, opportunity and ability to travel overseas for treatment.

Health New Zealand | Te Whatu Ora (Health NZ) briefly established a long COVID clinic in Christchurch; however, this clinic only ran for 5 months due to a lack of funding, staff shortages, and only being able to treat a limited number of people. There has been one long COVID service operating at Taranaki hospital since 2022; this clinic was initially set up for staff with long COVID [42, 43] although people with long COVID in the community are supported by the same allied health staff. The clinic does not receive funding, relying on Health NZ staff to incorporate the additional workload into their already busy lists. While long COVID clinics are ideal, they may not be pragmatic, furthering a reliance on the primary health care system of Aotearoa New Zealand to address long COVID.

Approaches to long COVID rehabilitation should be accessible, multi‐disciplinary, flexible, and person‐centred [44]. The implications for long COVID healthcare in Aotearoa New Zealand are clear [45, 46]. The Office of the Chief Science Advisor has noted that these symptoms deserve acknowledgement, attention, and dedication of health care resources [8]. There is a need to recognise long COVID as a disability and integrate services across primary and secondary care, and social support systems; wraparound, multidisciplinary care is vital. Primary health care providers require support for ongoing long COVID education and associated care pathways.

This study is part of a broader investigation that employs a co‐design partnership model involving those with lived experience of long COVID and health professionals working with researchers to develop a blueprint that outlines the core components of optimal long COVID management in primary health care. The fundamental tenet to this approach is placing people with lived experience of long COVID at the centre of the service design processes.

## 5 Conclusion

People living with long COVID in Aotearoa New Zealand face systemic, financial, and social barriers to care. A coordinated, compassionate, and evidence‐informed response is essential. Co‐design approaches, centring lived experience, must underpin future service development to ensure healthcare equity.

## Author Contributions

**Fiona McKenzie:** conceptualisation, formal analysis, funding acquisition, investigation, methodology, validation, writing – original draft. **Bailey Yee:** data curation, formal analysis, investigation, methodology, project administration, validation, writing – original draft, writing – review and editing. **Rochelle Ellison‐Lupena:** formal analysis, investigation, methodology, writing – review and editing. **Ridvan Firestone:** conceptualisation, formal analysis, funding acquisition, investigation, methodology, supervision, writing – review and editing. **Lis Ellison Loschmann:** conceptualisation, investigation, funding acquisition, methodology, writing – review and editing, formal analysis, supervision. **Kim van Wissen:** formal analysis, investigation, validation, writing – review and editing. **Mona Jeffreys:** conceptualization, formal analysis, funding acquisition, investigation, methodology, project administration, supervision; writing – review and editing.

## Ethics Statement

Ethical approval for the study was given by Victoria University of Wellington Human Ethics Committee 2023/HE312492. All participants gave written, informed consent prior to participation.

## Conflicts of Interest

The authors declare no conflicts of interest.

## Data Availability

Research data are not shared, as consent for this was not obtained from participants.
